# Sub-picosecond biphasic ultrafast all-optical switching in ultraviolet band

**DOI:** 10.1515/nanoph-2024-0415

**Published:** 2024-10-24

**Authors:** Xiaoxiang Dong, Yonglin He, Tao Zhu, Renxian Gao, Lingyun Hu, Jiayu Li, Peiwen Ren, Jian-Feng Li, Ming-De Li, Zhilin Yang

**Affiliations:** College of Physical Science and Technology, State Key Laboratory of Physical Chemistry of Solid Surfaces, Xiamen University, Xiamen 361005, China; Department of Chemistry and Key Laboratory for Preparation and Application of Ordered Structural Materials of Guangdong Province, Shantou University, Shantou, Guangdong 515063, P.R. China; College of Chemistry and Chemical Engineering, State Key Laboratory of Physical Chemistry of Solid Surfaces, Xiamen University, Xiamen 361005, China

**Keywords:** ultrafast, all-optical switching, ultraviolet, biphasic

## Abstract

Ultrafast all-optical control has been a subject of wide-spread attention as a method of manipulating optical fields using light excitation on extremely short time scales. As a fundamental form of ultrafast all-optical control, all-optical switching has achieved sub-picosecond switch speeds in the visible, infrared, and terahertz spectral regions. However, due to the lack of suitable materials, ultrafast all-optical control in the ultraviolet range remains in its early stages. We demonstrate sub-picosecond all-optical switching in the ultraviolet wavelength by designing a Si_3_N_4_-ITO Fabry–Pérot resonance aligns with the edge of the interband transition region of ITO. The response time of 500 fs achieved at a pump fluence as low as 0.17 mJ/cm^2^. Notably, unlike conventional binary switches (0, 1), this biphasic all-optical switch enables the modulation of optical intensity with positive, zero, and negative Δ*R*/*R* (0, 1, −1) at the same wavelength, all achieved with a switching speed of 680 fs at a pump fluence of 0.45 mJ/cm^2^. This work establishing a new pathway for all-optical control in the ultraviolet spectrum, the biphasic switch provides an extra degree of freedom for all-optical modulation.

## 1 Introduction

Ultrafast all-optical switching is a fundamental component for optical field manipulation, achieved by altering the optical response of the material through interactions with pulsed light. Due to its unparalleled speed and potential for on-chip integration, it is widely used for controlling the intensity, polarization, and phase of light [1], [2], [3], [4], [5], [6], [7], [8], [9]. The research on all-optical switching can be traced back to the optical double-stability experiment in 1976 [10]. Since its inception, there has been significant attention devoted to improving the response speed and expanding the operational wavelength range of all-optical switchings [11], [12]. To date, researchers have conducted extensive research on all-optical switchings in the visible light range utilizing plasmonic structures with metals, as well as semiconducting materials such as silicon and gallium phosphide [13], [14], [15], [16], [17], [18]. In the infrared spectrum, transparent metal oxides have been employed [19], [20], [21], [22], [23], [24], [25], [26], while in the terahertz range, investigations have been carried out using two-dimensional materials like graphene and transition metal sulphides [27], [28], [29], [30], [31]. Leveraging ultrafast third-order nonlinear processes such as optical Kerr effect (OKE) and two-photon absorption (TPA), the response speed of the aforementioned optical switchings in these spectral ranges has achieved sub-picosecond temporal scales [32], [33].

The ultraviolet band is crucial for studying biological and chemical molecules, as well as wide-bandgap semiconductors, playing an indispensable role in high-resolution imaging, lithography, and sensing [34], [35], [36]. High-speed optical field manipulation aids in better understanding and controlling the dynamic processes of these materials. Recently, the ultrafast dynamic processes and all-optical control of materials in the ultraviolet band have garnered increasing attention, following the work of Korff Schmissing et al., who achieved an ultrafast all-optical magnetic switch in the extreme ultraviolet band using the demagnetization dynamics of CdFe, with switching speeds on the order of hundreds of picoseconds [37]. However, the current sub-picosecond all-optical switchings in the ultraviolet spectrum remain unexplored due to challenges such as the slow response of material intrinsic bandgap transitions, a lack of effective control mechanisms, and difficulties in micro-nanofabrication at sub-300 nm periodic scales.

Indium tin oxide (ITO), as a representative of transparent conductive oxides (TCOs), has been extensively employed as ultrafast all-optical switchings in the infrared spectrum since Israel De Leon and others revealed its sub-picosecond ultrafast nonlinear processes in the epsilon-near-zero (ENZ) regime in 2016 [19], [38], [39]. Although TCOs have demonstrated fascinating performance in the infrared spectrum, there has been a lack of research exploring their potential for all-optical control in the ultraviolet band. We have noticed that the smaller electron heat capacity and lower carrier density in the conduction band of ITO still exist in the near-ultraviolet bands [40]. These characteristics enable ITO to be excited and relaxed in an extremely short time under intraband pumping, which is the key to achieving sub-picosecond response speed at the microscopic level [23]. And its approximately 3.7 eV bandgap in the ultraviolet band also provides the possibility for the implementation of ultraviolet all-optical switching [41].

Here in, we proposed and prepared a Si_3_N_4_-ITO reflective thin-film nanocavity, aiming to extend the operational wavelength of the all-optical switching into the ultraviolet range by superimposing Fabry–Pérot (FP) resonance and ITO interband transition modes. The nanocavity composed of Si_3_N_4_ and ITO enhances the coupling of light into the entire system. The low reflectance (*R*) of the FP resonance in the nanocavity’s spectrum provides a foundation for larger changes in reflectance (Δ*R*/*R*) induced by optical excitation, allowing the all-optical switching to operate at lower power levels [42], [43]. Utilizing transient absorption spectroscopy, we obtained an ultrafast response time of 500 fs for the thin-film nanocavity in the ultraviolet wavelength range. It is noteworthy that, without introducing optical parameters such as phase and polarization, we achieved transient reflectance modulation at a wavelength of 330 nm for the biphasic (Δ*R*/*R* > 0, Δ*R*/*R* = 0 & Δ*R*/*R* < 0) switching solely by designing the film thickness of Si_3_N_4_. This sub-picosecond biphasic modulation presents significant potential for enhancing the multidegree-of-freedom manipulation of optical fields. It is anticipated that other TCOs could leverage this approach to further extend the capabilities of all-optical control in the ultraviolet band.

## 2 Models and principles

**Figure 1:**
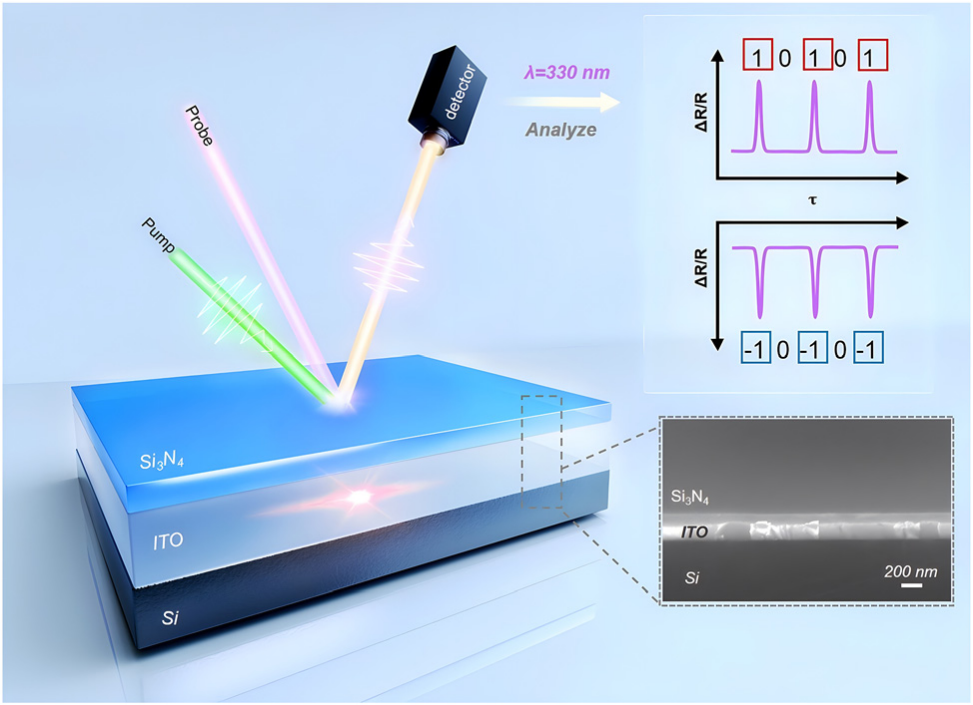
Concept diagram and SEM image of biphasic ultrafast all-optical switching. Left section represents the all-optical switch model, top right illustrates the biphasic switching principle, and the bottom right section shows SEM characterization images.

Figure 1 illustrates a schematic model of the ultraviolet biphasic all-optical switching, where the nanocavity is composed of a 175 nm thickness ITO film on a silicon substrate and top cover layers of 80 nm/100 nm Si_3_N_4_. Considering the bandgap of ITO, 550 nm was chosen as the pump wavelength (detailed analysis is discussed in Supplementary Information Section 4), while the probing light spans from 300 nm to 700 nm in continuous spectrum. The right section of Figure 1 depicts schematic representations of time-resolved reflectance signals at 330 nm wavelength for different thicknesses (80/100 nm) of Si_3_N_4_ cover layer. Transient reflectance spectra with Δ*R*/*R* > 0 are defined as “1” those with Δ*R*/*R* < 0 as “−1” and the reflection signal without pump (Δ*R*/*R* = 0) is considered as “0”. We define biphasic switching as the capability of the optical switch to achieve three distinct states (0, 1, and −1) – at the same wavelength and switching speed. The illustration presents a scanning electron microscope (SEM) image from a 45° top-down perspective. The ITO thin film is prepared on a silicon substrate using magnetron sputtering, while the Si_3_N_4_ layer is grown on the ITO using plasma-enhanced chemical vapor deposition (see methods for details). The two materials exhibit good compatibility and smoothness, making them suitable for integration with chip and CMOS processes [44] (as demonstrated in Supplementary Figure S1 with AFM and crystal XRD images). The role of Si_3_N_4_ extends beyond protecting the ITO layer and regulating cavity length, it also facilitates impedance matching for improved optical coupling throughout the system. Contrastingly, the comparison sample without a Si_3_N_4_ layer is demonstrated in Supplementary Figure S3b. Moreover, the absence of free electrons and a substantial bandgap width in Si_3_N_4_ ensures the avoidance of introducing inherent signals from the material itself. The refractive indices of ITO and Si_3_N_4_ in this study were obtained through spectroscopic ellipsometry measurements, and they are consistent with the relevant parameters reported in the literature [19], [45].

## 3 Ultraviolet anomalous transient reflection

**Figure 2:**
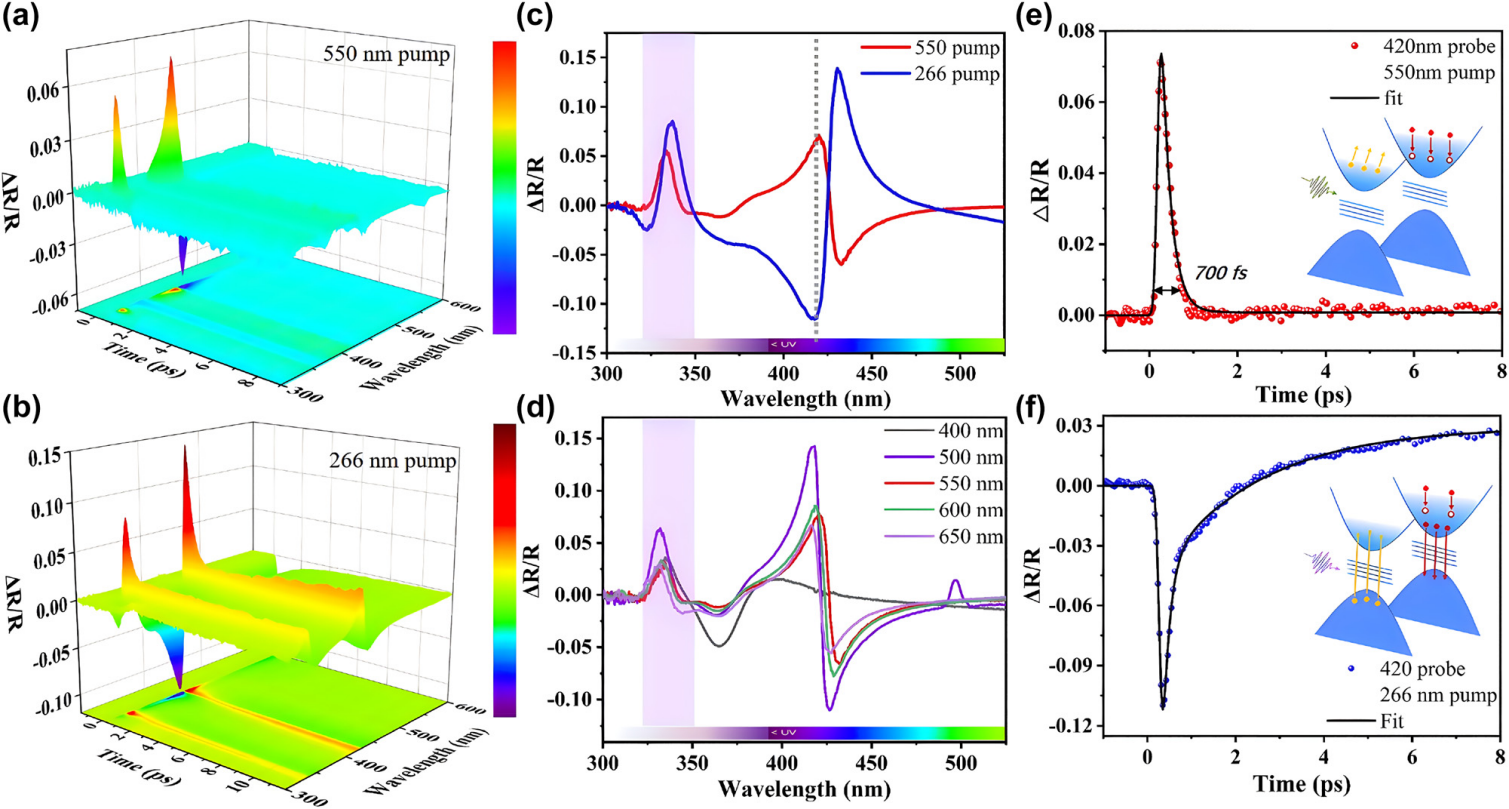
Transient optical measurements. (a) The time resolution Δ*R*/*R* spectral map under pump wavelength of 550 nm. (b) The time resolution Δ*R*/*R* spectral map under pump wavelength of 266 nm. (c) Δ*R*/*R* spectrum at 266 nm and 550 nm pump. (d) Pump wavelength dependence Δ*R*/*R* spectrum (pump fluence of 0.43 mJ/cm^2^). (e) Δ*R*/*R* kinetics corresponding to 550 nm pump and probe at 420 nm. (f) Δ*R*/*R* kinetics corresponding to 550 nm pump and probe at 420 nm. The electronic structure and electron processes of ITO under two pump methods are shown in the inset.

The time-resolved ultrafast spectroscopy technique is employed to investigate the biphasic all-optical switching. Figure 2a and b present the 3D plots of time-resolved transient reflectance spectra excited at wavelengths of 266 nm (with a pump fluence of 0.17 mJ/cm^2^) and 550 nm (with a pump fluence of 0.43 mJ/cm^2^). These pump wavelengths correspond to the intraband excitation and interband transition excitation of ITO, respectively (the specific reason for choosing the pump wavelength can be found in Supplementary Information Section 4). Despite differences in transient reflectance and response time upon the two pumping conditions, their resonance wavelength positions (330 nm and 425 nm) are consistent. This implies that resonance in the transient ultraviolet spectrum can be achieved by constructing a Si_3_N_4_–ITO nanocavity. Figure 2c shows Δ*R*/*R* transient spectra upon 266 nm and 550 nm pump, respectively, extracted from the Figure 2a and b, here, the chosen delay time is that when Δ*R*/*R* reached a maximum (at approximately 0.26 ps). Under the 266 nm wavelength pump, the transient reflection spectrum exhibits adjacent valley and peak features at 425 nm, along with similar patterns at 330 nm (depicted by the blue line). When subjected to a 550 nm wavelength pump, the transient reflection spectrum displays adjacent peak and valley features at 425 nm, while at 330 nm, it manifests as an isolated peak (depicted by the red line). Notably, under 550 nm excitation, the isolated peak observed at 330 nm is distinct from other scenarios, representing the superposition of the Fabry–Pérot cavity modes and interband transition modes (details of which will be elaborated in subsequent sections and Supplementary S10). The transient reflection spectrum, characterized by consecutive peaks and valleys, corresponds to the shift in the dip position of the steady-state reflection spectrum (associated with the Fabry–Pérot resonance, to be analyzed and discussed later) induced by the pump. The movement of this reflection dip position is attributed to changes in the permittivity stimulated by the pumped light, as described in Equation (1).

(1) $$\begin{array}{ll} \varepsilon \left(\omega \right) & =\varepsilon_{b}\left(\omega \right)+\varepsilon_{f}\left(\omega \right)=\varepsilon_{b}\left(\omega \right)-\frac{\omega_{p}^{2}}{\omega \left(\omega +i\Gamma \right)}\\ \omega_{p} & =\sqrt{n_{e}e^{2}/\varepsilon_{0}m_{e}^{*}} \end{array}$$

where *ω*_*p*_ is the plasma frequency, *n* represent the electron density, *e* is the electron charge, *ε* is the vacuum absolute permittivity, and *m*_eff_ is the effective electron mass [46]. The intricate influence of interband transitions and intraband pumping on the permittivity is elaborated in Supplementary Information Section 4. Two distinct pumping methods resulted in different trends in the permittivity, causing a transformation at the 425 nm wavelength in the transient reflection spectrum. However, this effect was not prominently observed in the ultraviolet range, suggesting additional processes at play in the resonance at 330 nm. To comprehend the kinetic processes of interference caused by the superposition of detection light at 330 nm, inducing interband transitions, one can initially investigate the kinetic processes of detection light at 420 nm wavelength, which does not involve interband transitions. Figure 2e and f depict the kinetics at 420 nm wavelength corresponding to 550 nm and 266 nm pumping. In the case of intraband pumping, an ultrafast response with a time scale of approximately 700 fs is observed, while interband pumping corresponds to a longer relaxation process (which can extend into the microsecond range due to lattice cooling, as illustrated in the complete kinetics in Supplementary Figure S6). This means that the intraband pumping method is more suitable as the excitation wavelength for ultrafast all-optical switchings. The pump wavelength dependency of the ultrafast all-optical switching is illustrated in Figure 2d. It can be observed that when the pump wavelength exceeds 500 nm, the Δ*R*/*R* spectra exhibit remarkable similarity, characterized by an isolated peak in the ultraviolet region. This observation indicates that, at a pump fluence of 0.43 mJ/cm^2^, the appearance of the ultraviolet isolated peak is not solely dependent on a specific excitation wavelength. Additionally, it reveals a consistent trend of decreasing peak intensity with increasing wavelength.

## 4 Biphasic ultrafast all-optical switching

**Figure 3:**
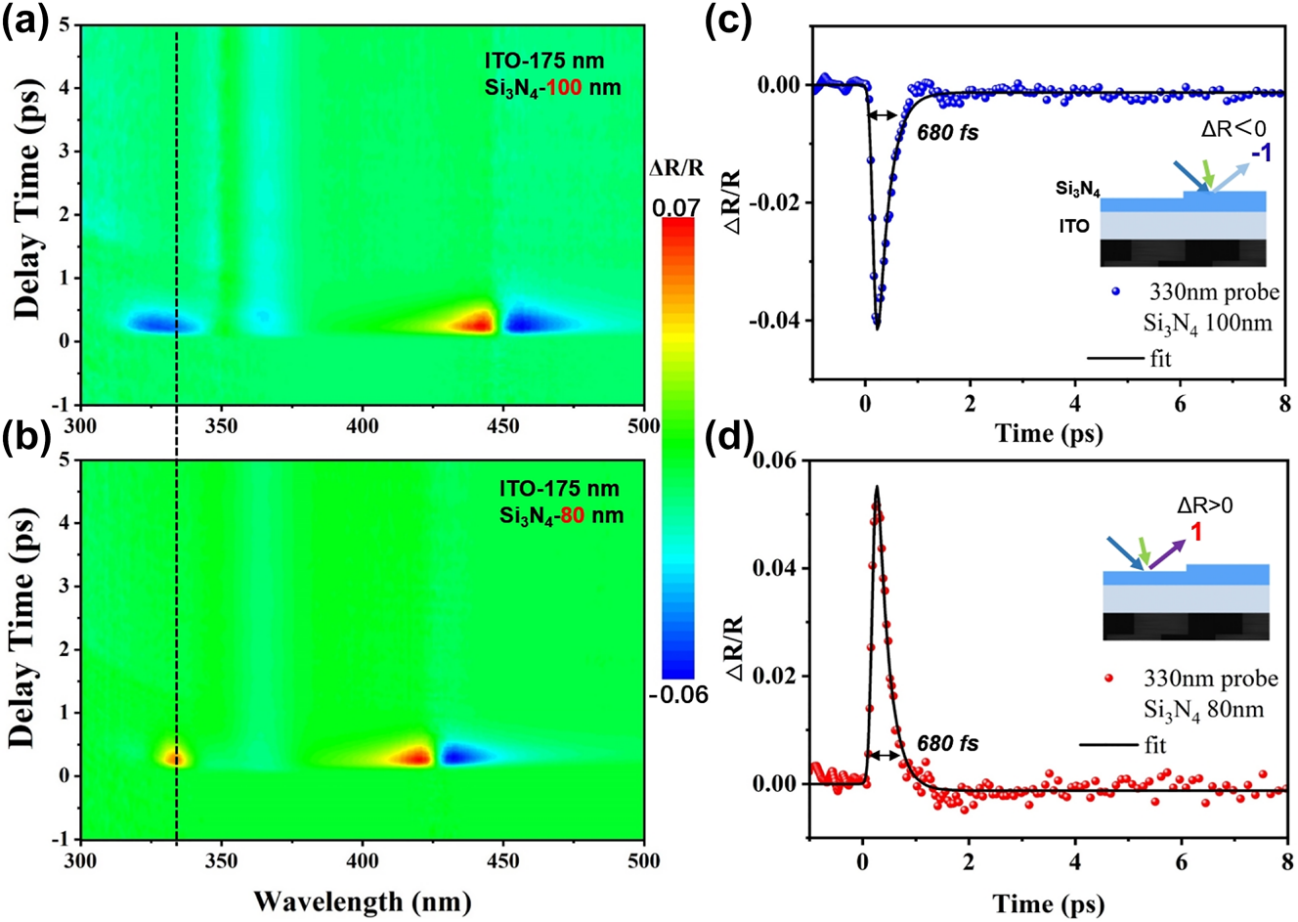
Transient characterization of the biphasic ultrafast all-optical switching. (a) The time resolution Δ*R*/*R* spectral map of Si_3_N_4_-ITO 100 nm–175 nm. (b) The time resolution Δ*R*/*R* spectral map of Si_3_N_4_-ITO 80 nm–175 nm. (c) The Δ*R*/*R* kinetics at 330 nm of Si_3_N_4_-ITO 100 nm–175 nm. (d) The Δ*R*/*R* kinetics at 330 nm of Si_3_N_4_-ITO 80 nm–175 nm. The pump center wavelength is kept at 550 nm, and pump fluences are 0.43 mJ/cm^2^, respectively.

Figure 2e and f demonstrate that under both interband and intraband pump conditions, Δ*R*/*R* at a wavelength of 420 nm exhibits opposite signs, enabling optical field manipulation on different time scales (sub-ps and µs). Similar interesting effects have been reported in previous studies [26], [30], [47]. However, the inability of both switching speeds to simultaneously reach the sub-picosecond range fundamentally distinguishes our ultrafast biphasic all-optical switching, discussed later in this work. We anticipate that the isolated peak in the transient reflectance spectrum within the ultraviolet range is exceptionally well-suited for application in an ultraviolet all-optical switching. Furthermore, this easily fabricated thin-film nanocavity structure allows for the creation of samples with varying thicknesses. Additionally, as shown in the inset of Figure 3c, two different thicknesses of Si_3_N_4_ can be prepared on the same ITO substrate, thereby enhancing the functionality of the all-optical switching. The time-resolved Δ*R*/*R* spectra for a set of samples with different thicknesses are illustrated in Figure 3a and b. Both samples have an ITO layer thickness of 175 nm, while the Si_3_N_4_ layers correspond to 100 nm and 80 nm, respectively. In fact, the choice of 80 nm and 100 nm thicknesses was based on a series of comparative experiments on different Si_3_N_4_ thicknesses, as shown in Supplementary Figure S8. While other thicknesses produced similar effects, the transient reflectance spectra for Si_3_N_4_ with thicknesses of 80 nm and 100 nm exhibited more pronounced characteristics. Under 550 nm pumping, in the visible region their spectra exhibit consistent positive and negative Δ*R*/*R* signals (depicted in red and blue), with the central wavelength shifting from around 450 nm to approximately 425 nm. However, in the ultraviolet response region, devices with different thicknesses display distinct positive or distinct negative signals (in red or blue), with opposite Δ*R*/*R* signs in their reflection spectra. Figure 3c and d, respectively, illustrate the dynamic processes of the two devices at a wavelength of 330 nm. It is evident that both exhibit switch time around 680 fs, with Δ*R*/*R* values at a similar magnitude of approximately 5 %, and their signs are precisely opposite.

In this context, two values of the Δ*R*/*R* transient reflection spectra at the same wavelength, with similar magnitudes but opposite signs and consistent switching times, are defined as biphasic. Therefore, the ultraviolet transient response of devices with two different thicknesses under intraband pumping can serve as a set of biphasic ultrafast all-optical switching. In Section 7 of the Supplementary Information, we perform additional comparative experiments with ITO of different thicknesses, demonstrating that ultrafast biphasic switching can also be achieved. This shows that the biphase switch does not depend on a specific ITO thickness, can be reproduced at other thicknesses, and that varying the ITO thickness can also be adjusted to a small range rather than just limited to a single wavelength of 330 nm. The biphasic all-optical switch not only exhibits sub-picosecond response in the ultraviolet region but also improves the ability of light field regulation by introducing additional degrees of freedom, allowing for free selection of the increase or decrease of reflectivity. It is worth noting that the positive and negative symbol switching ability of the biphasic optical switch is more intuitive than the traditional optical switch that only observes the change of reflectivity, so it is not necessary to rely on high pump flux to increase the modulation depth.

## 5 Discussion

**Figure 4:**
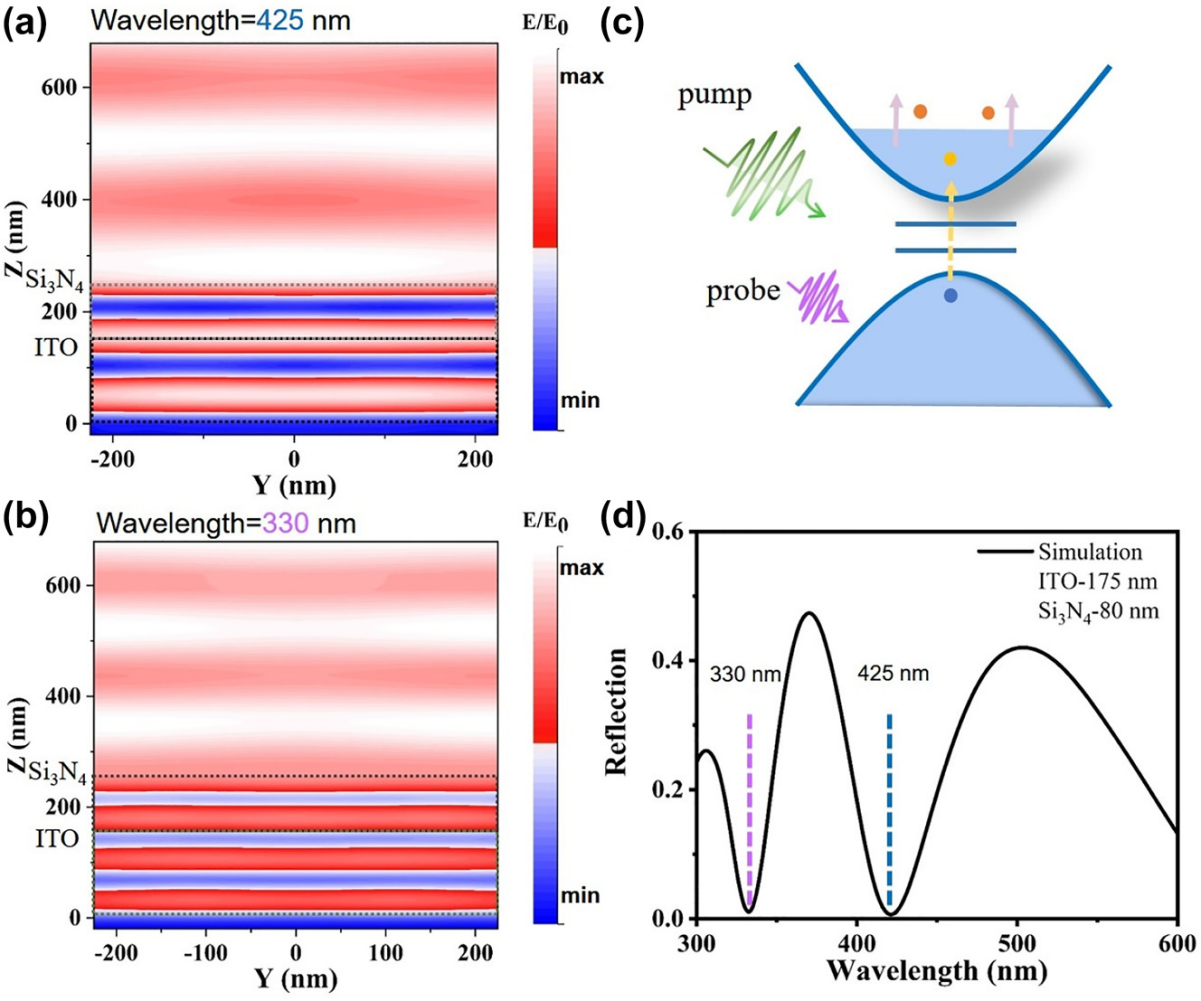
Simulation and analysis of ultrafast all-optical switching. (a) The simulated electric field intensity distribution of the nanocavity at 425 nm. (b) The simulated electric field intensity distribution of the nanocavity at 330 nm. (c) Schematic diagram showing the electron configurations and electronic processes involved in the 550 nm intraband pumping and ultraviolet probe. (d) Simulation static reflection spectra of Si_3_N_4_-ITO.

In order to further clarify the intrinsic mechanism of the working principle of optical switching, the electric field distribution within the nanocavity at wavelengths 425 nm and 330 nm, obtained from FDTD simulations (see methods for details), is presented in Figure 4a and b. The region of *z* = 0–175 nm corresponds to the ITO layer, while *z* = 175–255 nm represents the Si_3_N_4_ thin-film region. Both configurations exhibit typical F–P cavity modes, with positions of enhanced field strength within the ITO layer. Figure 4d shows the simulated reflection spectra, where the F–P resonance at 330 nm and 425 nm exhibit a reflection dip close to zero, aligning with the positions in the transient reflection spectra. It can be inferred that the peak and valley near 425 nm in the transient reflection spectrum are caused by a redshift in the F–P resonance due to changes in the permittivity of ITO induced by the pump light. It could be attributed to a change in permittivity induced by hot electrons, with the timescale being governed by electron–phonon coupling. A shift in the FP resonance is evident near 330 nm as well. However, at 330 nm, the detection wavelength reaches the bandgap edge of ITO, and the isolated peak at 330 nm is attributed to the combined effect of the FP cavity mode and interband transitions. The absorption in the ITO’s valence band below 340 nm, inferred from the imaginary part of the dielectric function in Supplementary Figure S5, indicates that electrons in the ITO valence band below 340 nm wavelength undergo transitions to the conduction band. In the model depicted in Figure 4c, the photon energy of the probing light is absorbed to excite electron transitions from the valence band to the conduction band. This leads to a slight increase in the electron concentration in the conduction band, participating in the intraband excitation process (a more intuitive spectral explanation can be found in Supplementary Figure S11). Since the power of the probing light is much lower than that of the pump light, this process does not induce significant thermal effects (its slow dynamics are hardly observable, likely due to cancellation with the weak reflectivity of the Si substrate). Due to the low electron heat capacity of ITO and the electron-energy-loss-rate between electrons and the lattice, the dynamics of ITO occur on a sub-picosecond timescale. Considering that the silicon substrate also has a response at 330 nm, albeit a weak one, and that the sign of Δ*R* appears to be opposite, its presence cancels out part of ITO’s slower processes, allowing the switching speed to reach 680 fs.

**Figure 5:**
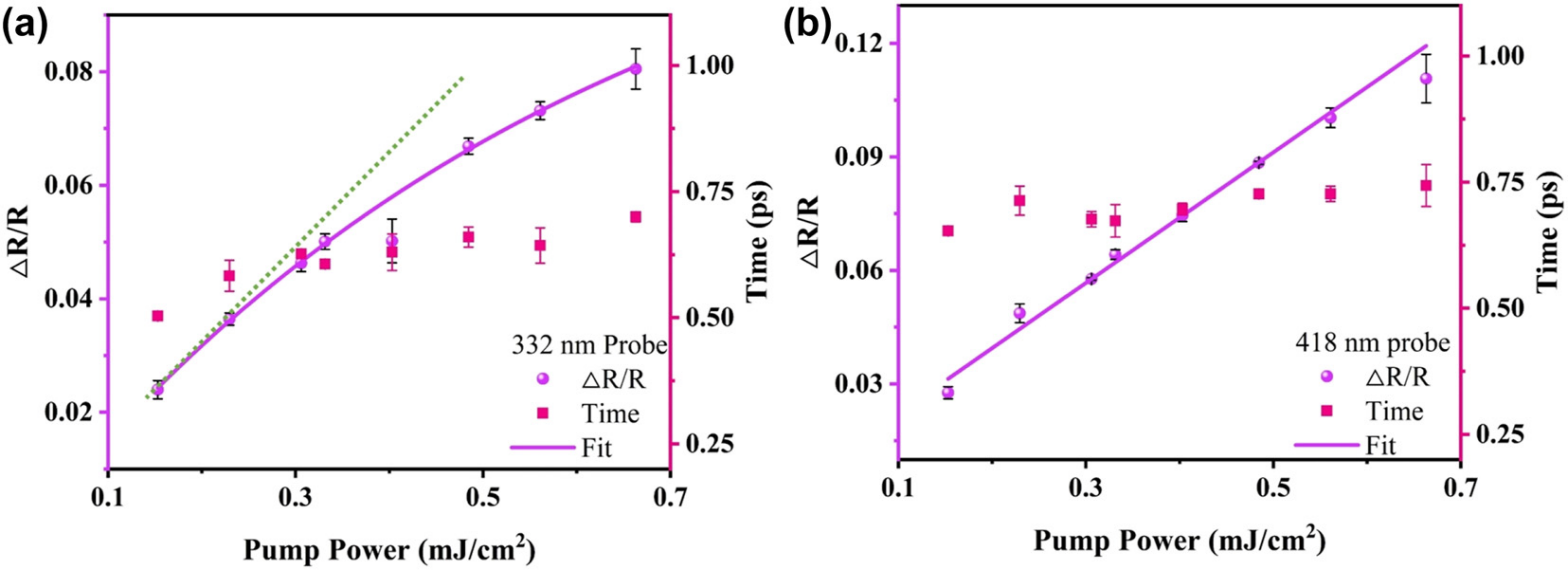
The pump fluence dependence of all-optical switching. (a) Dependence of modulation depth of Δ*R*/*R*(Max) and switching speed on pump fluence at ultraviolet band. (b) Dependence of modulation depth of Δ*R*/*R*(Max) and switching speed on pump fluence at visible band. The pump center wavelength is kept at 550 nm, respectively.

Finally, we investigated the pump fluence dependence of all-optical modulation depth and response speed in both the ultraviolet range and the contrasting visible range. Figure 5 reports the dual *y*-axes representing Δ*R*/*R* modulation depth and switching speed. Upon 550 nm wavelength pump, as the power density increases from 0.17 to 0.7 mJ/cm^2^, the increase in Δ*R*/*R* at the ultraviolet region follows a saturated nonlinear trend, rising from 2 % to 8 %. The response time experiences a slight increase, ranging between 500 fs and 740 fs. In contrast to the nonlinear growth in the ultraviolet range, the modulation depth in the visible region linearly increases with pump fluence, as depicted in Figure 5b. The switching speed experiences a slight increase from 620 fs to 750 fs. These difference of Δ*R*/*R* variation trends is attributed to the fact that, in the visible region, only electrons in the conduction band participate in intraband pumping excitation, whereas in the ultraviolet region (330–332 nm), interband transitions, correlated with the probing light, remain unaffected by the increase in pump light fluence. The distinct trends at the two wavelengths further support the notion that the ultraviolet region involves an additional mode, namely the contribution of interband transition behavior. We also explored the scenario under 266 nm wavelength pumping in our Supplementary Information Section 8, and the findings closely align with those observed under 550 nm pumping. The anomalous features of this ultraviolet region in transient spectra also manifest temperature dependence. In Supplementary Information Section 9, we investigated its behavior at temperatures ranging from 77 K to 310 K, revealing its potential application in temperature sensing. Therefore, the ultraviolet biphasic switching can not only leverage its multidegree-of-freedom advantage for applications in optical communications and quantum computing but also be directly utilized for low-temperature sensing.

## 6 Conclusions

In conclusion, we have achieved, for the first time, the sub-picosecond all-optical switchings operating in the ultraviolet wavelength range by employing ITO-Si_3_N_4_ thin-film nanocavities. Through the combination of Fabry–Pérot cavity resonance and ITO’s interband transition, the thin film nanocavity achieves biphasic switching with three degrees of freedom (1, 0, −1) at 330 nm, showcasing positive, zero, and negative Δ*R*/*R* values. The device exhibits a rapid response time of 500 fs under intraband pumping with a minimal pump fluence of 0.7 mJ/cm^2^. The biphasic all-optical switching expands the scope of ultrafast switchings, offering a broader platform for applications in all-optical control and beyond. It is anticipated that this method can be extended to other TCOs materials to achieve ultrafast all-optical switching at a wider range of different wavelengths.

## Supplementary Material

Supplementary Material Details
