# Supplementary material for: Sub-picosecond biphasic ultrafast all-optical switching in ultraviolet band
Source: Nanophotonics. 2024 Oct 24;13(24):4451–8. doi: 10.1515/nanoph-2024-0415 (PMC11636473; doi:10.1515/nanoph-2024-0415)
Supplement: Supplementary file 1 — Supplementary Material Details [file j_nanoph-2024-0415_suppl_001.docx]

Supporting Information

Biphasic Ultrafast All-optical Switching in Ultraviolet Band

*Xiaoxiang Dong, Yonglin He, Tao Zhu, Renxian Gao, Lingyun Hu, Jiayu Li, Peiwen Ren, Jian-Feng Li, Ming-De Li*, Zhilin Yang**

Methods

*Time-Resolved Experiments.* The ultrafast dynamics of biphasic all-optical switching were monitored using femtosecond pump−probe spectroscopy. An 80-fs amplified titanium sapphire laser (Solstice, Spectra-physics) served as the pump source. The wavelength of the pump source was tuned to 550 nm by the optical parametric amplifier (OPA-800CF, Spectra-physics) with a harmonic’s module. A variable ND filter was added to regulate the pump power. The collimated pump beam with a Gaussian diameter of 1 mm was employed to excite the vibration modes of samples at a 15° incidence angle. The probe beam, in this case, originated from the CaF Crystal lens (with λpr = 310−600nm). A mechanical chopper operating at 1 kHz modulated the pump power. Pump-induced relative changes of the probe beam reflection, ΔOD, were measured using synchronous detection as a function of the time interval separating pump and probe pulses, controlled by a mechanical delay line. The schematic diagram of femtosecond transient reflection spectroscopy is presented in Supporting Information Section 2.

*Numerical Simulations.* Simulations were carried out by a commercial software package (Lumerical Company) that adopted the 3D-FDTD method. The refractive indices and thicknesses of the ITO and Si3N4 layers were obtained through ellipsometry measurements and subsequent fitting. Subsequently, these parameters were input into the material module of the finite-difference time-domain (FDTD) simulation. The light source configuration was adapted to incorporate a 15-degree oblique incidence, maintaining polarization along the x-axis through the utilization of a plane wave. Throughout the simulation procedure, a perfectly matched-layer boundary condition was applied along the z-direction, while periodic boundary conditions were implemented along the x- and y-axes. To ensure convergence and accuracy, the simulation time was set to 1000 fs, and the Yee-cell size was configured to 1 nm. Finally, the enhancement of the electromagnetic field was post-processed using E/E_0_), where E and E_0_ denote the localized electric field and the incident electric field, respectively.

**S1.Preparation and characterization of structures**

The Si_3_N_4_ film is deposited through Plasma-Enhanced Chemical Vapor Deposition (PECVD). The process commences by establishing the chamber at a baseline vacuum below 5×10-5 Pa to minimize contamination risks. The substrate is preheated to 130°C. Gas flow initiates with 5% silane in 95% helium at 145 sccm, argon at 140 sccm, and ammonia at 8 sccm. The chamber pressure stabilizes at 5 Pa. With an RF power of 350 W, the deposition progresses at a rate of 367 seconds per 100 nm, ensuring consistent and controlled film growth.

The ITO films are fabricated through DC reactive magnetron sputtering, utilizing a finely crafted ITO target comprising a 90:10 ratio of In_2_O_3_ to SnO_2_, with an impressive purity of 99.99%. This precise Sn doping is crucial for enhancing the ITO's electrical properties without compromising its optical transmittance. Employing a 4-inch diameter target, the process maintains a target-to-substrate distance of 10 cm. The sputtering chamber reaches a high vacuum of below 3×10-6 Torr to ensure utmost purity and avoid contamination. Gas flow rates are meticulously controlled, with Argon at 20 sccm and Oxygen at 0.6 sccm. The DC power is set at 300 W, and each deposition phase lasts 210 seconds, meticulously laying down a film with a thickness of about 100 nm. The substrate is subject to precise thermal and rotational controls to achieve the desired film properties.


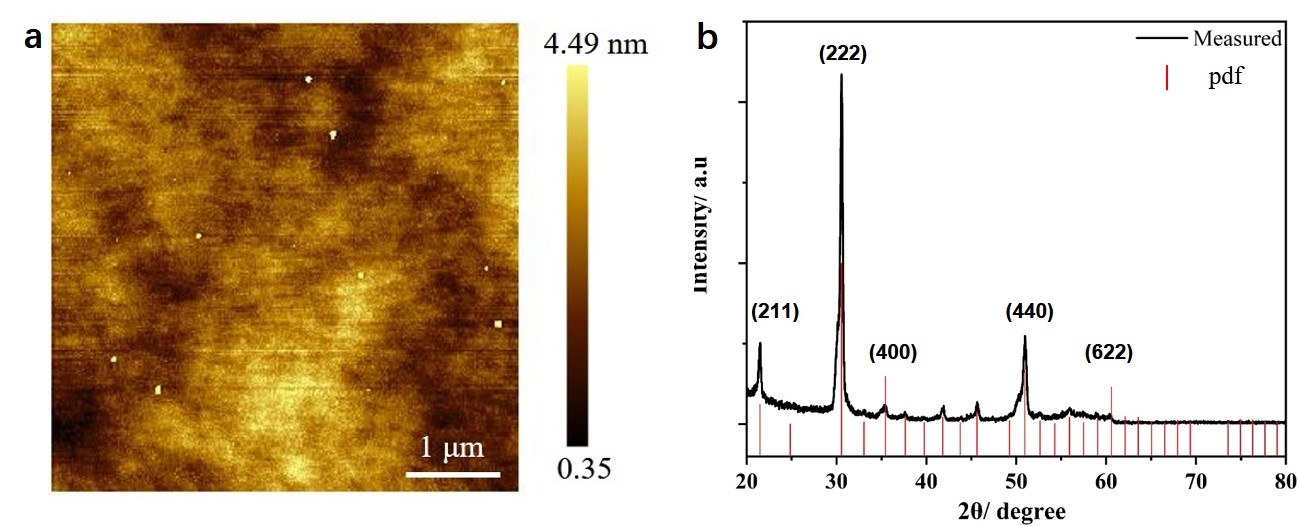


**Fig. S1.** Characterization of sample materials. a, AFM image of the top layer of Si_3_N_4_, with an RMS roughness of 579.1 pm. b, XRD diffraction spectrum of the sample.

**S2. Schematic diagram of the pump-probe experiment**


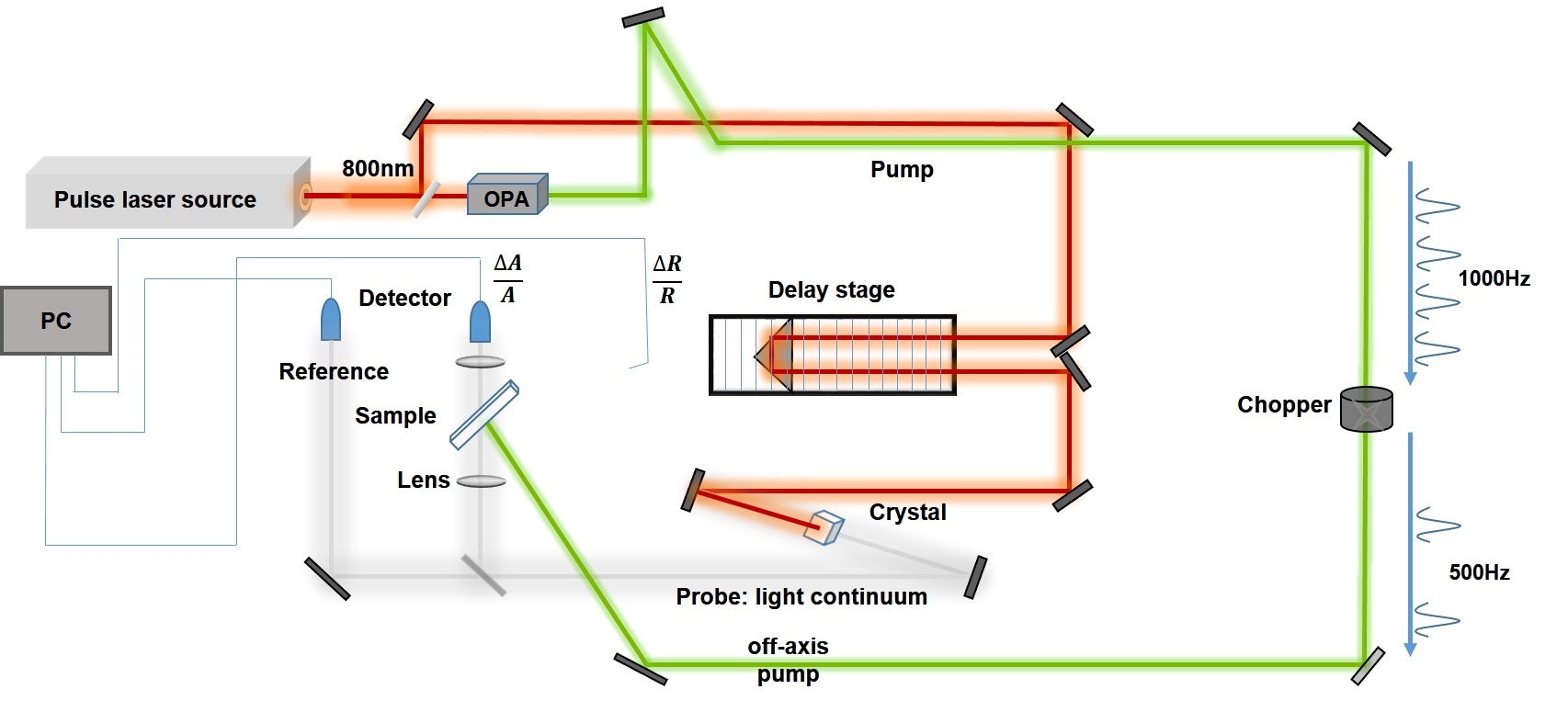


**Fig. S2**. Schematic diagram of the pump-probe experiment. Schematic showing the ultrafast pump-probe setup

**S3. Temporal dynamics of control group**

To comprehend the contributions in the transient reflectance spectra of each material in the multilayered structure, we characterized the time-resolved reflectance spectra of three control group samples: 1) silicon substrate, 2) ITO on silicon substrate, and 3) Si_3_N_4_ on silicon substrate. As shown in Supplementary Fig. S3a, Under 550 nm wavelength pumping (0.43 mJ/cm²), the silicon substrate exhibits a response with a peak center wavelength at 366 nm, represented by the blue region. This blue region corresponds to the intrinsic response position of the single-crystal silicon wafer, and signals at this position in other transient reflectance spectra throughout the main text are also attributed to the silicon substrate. In fact, the silicon substrate shows a response across the entire test wavelength range, with the maximum near 366 nm. The response time for this is greater than 50 ps.

In Supplementary Fig. S3b, a 100 nm-thick Si_3_N_4_ film is deposited on the silicon substrate, and its transient reflectance spectrum is very similar to that of the silicon substrate, showing no introduction of new rapid responses. Supplementary Fig. S3c displays a 175 nm-thick ITO film deposited on the silicon substrate, exhibiting a weak sub-picosecond response in the ranges of 400 nm-450 nm and 450 nm-500 nm. It can be inferred that the sub-picosecond component of the multilayer film system originates from ITO. However, samples lacking the top Si_3_N_4_ layer exhibit extremely low signal-to-noise ratios in the transient reflectance spectra at the same pump fluence, making them almost unobservable. This indicates that the high refractive index provided by the top Si_3_N_4_ layer plays a crucial role in coupling the pump light into the system.


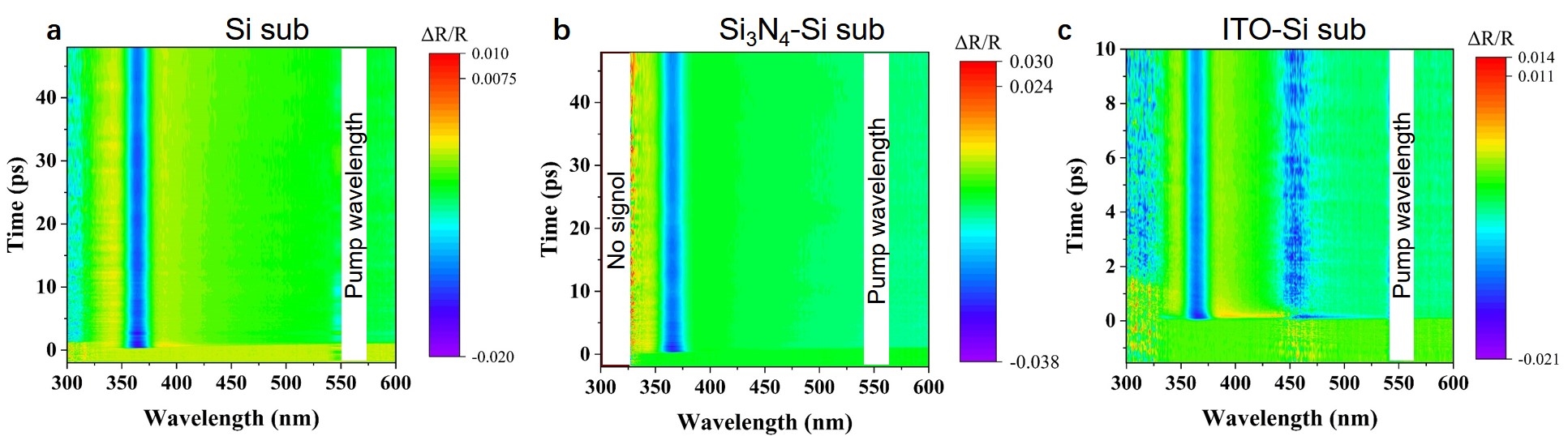


**Fig. S3** The pump-probe spectroscopy setup in reflectance mode with a 550 nm pump and 300 nm to 600 nm probe a, The time resolution ΔR/R spectral map of Si substrate. b, The time resolution ΔR/R spectral map of 100 nm Si_3_N_4_ on Si substrate. c, The time resolution ΔR/R spectral map of 100 nm ITO on Si substrate. The pump centre wavelength is kept at 550 nm and pump fluences are 0.43 mJ/cm2, respectively.

**S4. The control group samples under 266 nm pump and 550 nm pump**

For the selection of the pump wavelength, we considered that the 266 nm ultraviolet pump light would excite both the interband transition of ITO and the response of the single-crystal silicon substrate. Using plasma excitation of ITO requires a wavelength above 1240 nm, which cannot excite the response of the silicon substrate, making it an uncontrollable variable. In this context, we attempted to use visible light for pumping, such as the 550 nm wavelength. We found that it could excite both the interband transition of ITO and the response of the silicon substrate. Although ITO has minimal absorption in the visible range, the extremely low reflectance at the FP resonance enables the observation of changes in ITO's intra-band refractive index. The response of single-crystal silicon beyond 366 nm is weak, and the dynamics of lattice cooling in ITO overlap, canceling each other at certain wavelengths in the spectra. Therefore, what is observed in the transient reflectance spectrum is the superposition of almost only sub-picosecond components of these processes. Additionally, we observed that without the Si_3_N_4_ layer, the 550 nm wavelength almost couldn't excite the ITO film on the Si substrate (no Si_3_N_4_ layer), as shown in Supplementary Fig. S3c. While the 266 nm wavelength could excite the ITO film on the Si substrate (no Si_3_N_4_ layer), it exhibited features of interband transition in the spectrum below 330 nm, as seen in Supplementary Fig. S5a. The Si_3_N_4_ layer, which absorbs light below 320 nm, effectively filters out this interference signal in the Si_3_N_4_-ITO system.

Distinguishing between interband transition pump and intraband transition pump-induced changes in the permittivity of ITO can be identified by the variation pattern of ωp. As it is known $\omega_{p}^{2}=ne^{2}/m$, for a free electron gas, where n is the electron density, e the elementary charge and m the effective mass. Instead of being constant in a parabolic band, the effective mass in a non-parabolic band becomes k (the electron wave vector) -dependent and increases for higher energy states. For intra-band pumping, altering the effective electron mass (m) can be achieved by generating a hot electron distribution within its non-parabolic conduction band.[1] The timescale of m variation is on the order of hundreds of femtoseconds, determined by the rate of thermal exchange between electrons and the lattice. Under conditions of inter-band transition excitation, a significant number of electrons from the valence band transition to the conduction band, resulting in a notable increase in electron density (n) within the conduction band.[2] Modulation of ωp is achieved by increasing the electron density (n).

$$\omega_{p}{(\mu,T)}^{2}=\frac{e^{2}}{3m\pi^{2}}\int_{0}^{\infty} dE[\frac{2m}{\hbar^{2}}(E+E^{2}/E_{g})]^{\frac{3}{2}}$$

$$(1+2E/{E_{g}})^{-1}(-\frac{\partial f_{0}(\mu,T)}{\partial E})$$

$$n\left( \mu,T \right)=\frac{1}{\pi^{2}}\int_{0}^{\infty} dE\frac{m}{\hbar^{2}}(1+E/{E_{g}})$$

$$(\frac{2m}{\hbar^{2}}(E+{E^{2}}/{E_{g}}))^{\frac{1}{2}}f_{0}(\mu,T)$$

For samples with varying thicknesses of the same material Si_3_N_4_-ITO (80-220 nm), (100-175 nm), and the control group, we applied the same pumping conditions as in the main text: 0.43 mJ/cm² at 550 nm and 0.17 mJ/cm² at 266 nm. Supplementary Fig. S4 illustrates the transient reflectance spectra for the two control samples under these two pumping conditions. In Supplementary Fig. S4a, peaks and valleys near 375 nm and 500 nm experience a reversal in sign under both pump wavelengths, while the resonance around 325 nm maintains a consistent sign regardless of the pump wavelength. In Supplementary Fig. S4b, peaks and valleys near 450 nm change sign under both wavelengths, while the resonance around 340 nm remains unchanged. The two control group samples, along with the samples mentioned in the main text, exhibit consistent patterns around 330 nm, unaffected by intraband and interband pumping variations. Therefore, the anomalous phenomenon observed in the ultraviolet range in the main text is reproducible, and there is reason to believe that the unusual behavior of ITO around 330 nm is associated with its interband transitions.


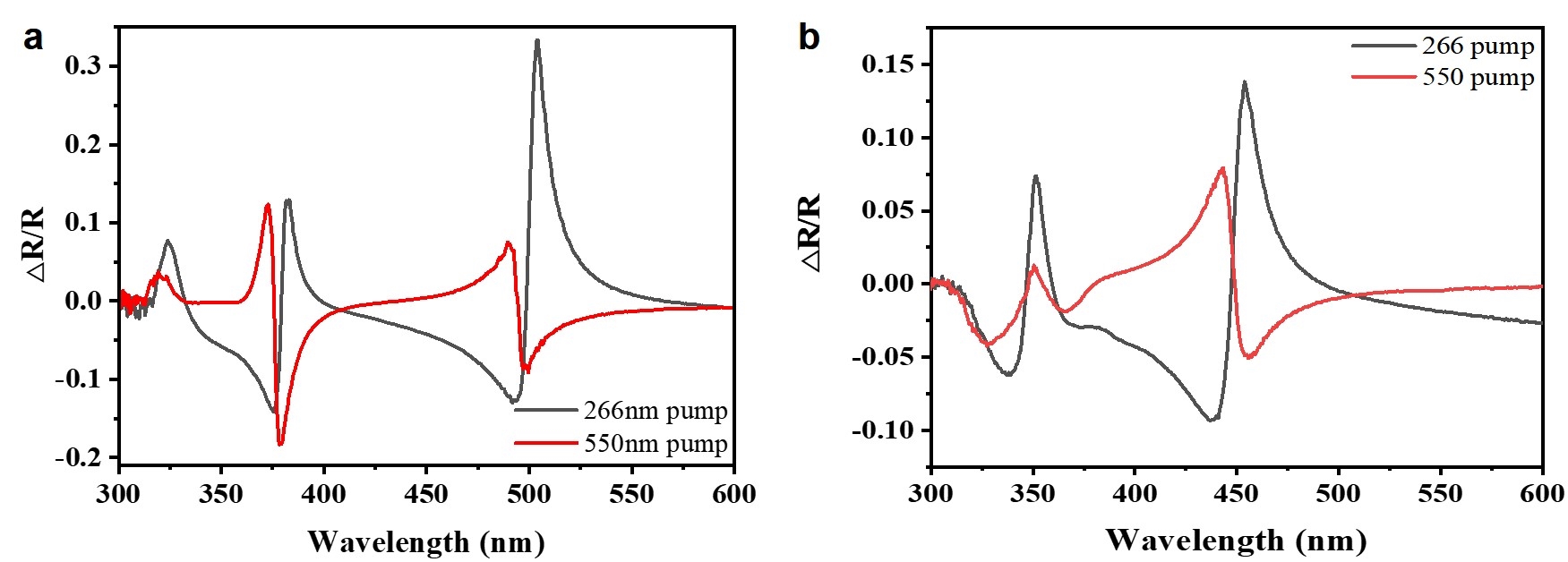


**Fig. S4.** The time-resolved reflectance spectra for the control group under two different pump wavelengths. a, 80nm Si_3_N_4_ 220 nm ITO. b, 100 nm Si_3_N_4_ 175 nm ITO.

**S5. Analysis and discussion of ITO at a wavelength of 330 nm**

Supplementary Fig. S5a depicts the transient reflectance spectra of 100 nm thick ITO on a silicon wafer under 266 nm pump conditions (0.255 mJ/cm²). This measurement was conducted to mitigate interference from Si_3_N_4_ in the system. In the range of 300 nm to 340 nm, we observed a positive region around 1.5 ps, while a negative signal appeared near 330 nm. This indicates that the high-energy 266 nm ultraviolet pump can couple into the ITO-Si system without relying on the Si_3_N_4_ layer. Moreover, there is a pronounced response at wavelengths below 340 nm, and around 460 nm, a connected peak and valley structure is evident. Supplementary Fig. S5b displays the fitting data of the real and imaginary parts of the Si_3_N_4_ -ITO permittivity obtained from ellipsometry. The imaginary part of ITO's permittivity significantly increases from 340 nm to 250 nm, corresponding to ITO's bandgap. Wavelengths below 340 nm can induce interband transitions in ITO. The steady-state simulation spectra of the sample are shown in the Supplementary Fig S5c, where the two FP resonances at 330 nm and 460 nm correspond to the resonant positions in the transient spectra presented in Supplementary Fig S5a.


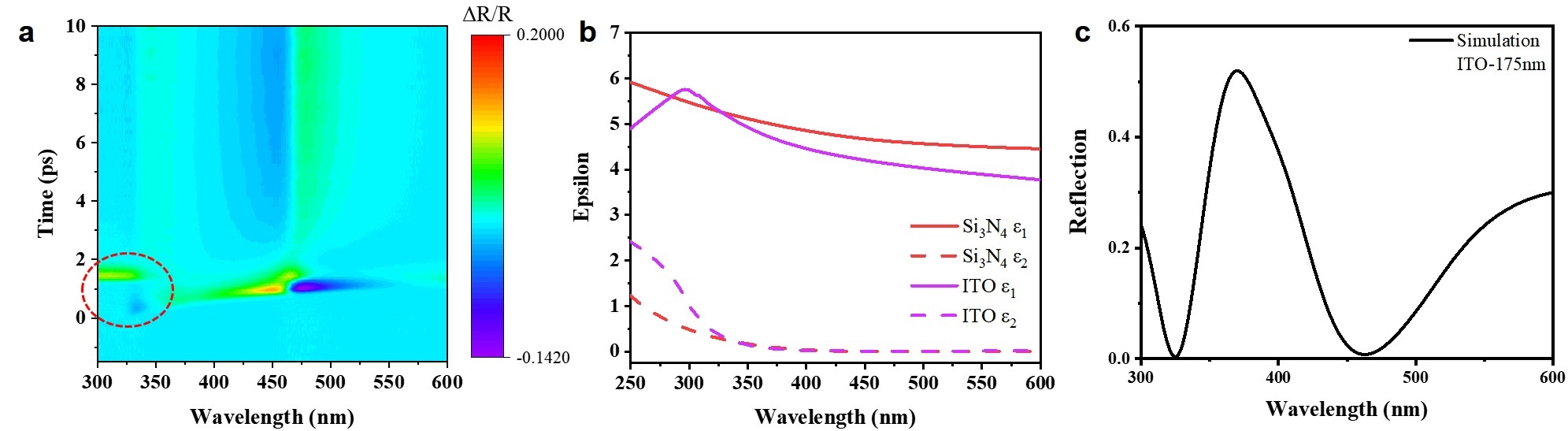


**Fig. S5.** The anomalous behavior of ITO in the ultraviolet region. a, The time resolution ΔR/R spectral map of ITO-Si b, The permittivity of ITO and Si_3_N_4_ films. c, Simulation static reflection spectra of Si_3_N_4_-ITO.

**S6. The microsecond component of kinetics**

To fully characterize the dynamics, we performed both picosecond TA experiments (denoted as short-delay-TA experiments) and microseconds TA experiments (denoted as long-delay-TA experiments). Supplementary Fig.S5a and b present the transient reflectance spectra for the 175 nm thick ITO and 80 nm/100 nm Si_3_N_4_ (i.e., the two samples representing the biphasic switch in the main text) under excitation at 266 nm wavelength (0.54 mJ/cm²) in long-delay transient absorption experiments. Both exhibit a relaxation time of approximately 0.1 microseconds, attributed to their slower lattice cooling. Under 550 nm excitation, the slow process is not observed, while the 266 nm interband transition excitation induces a pronounced thermal effect due to its higher energy. Previous literature has also discussed the lattice cooling of ITO.[3]


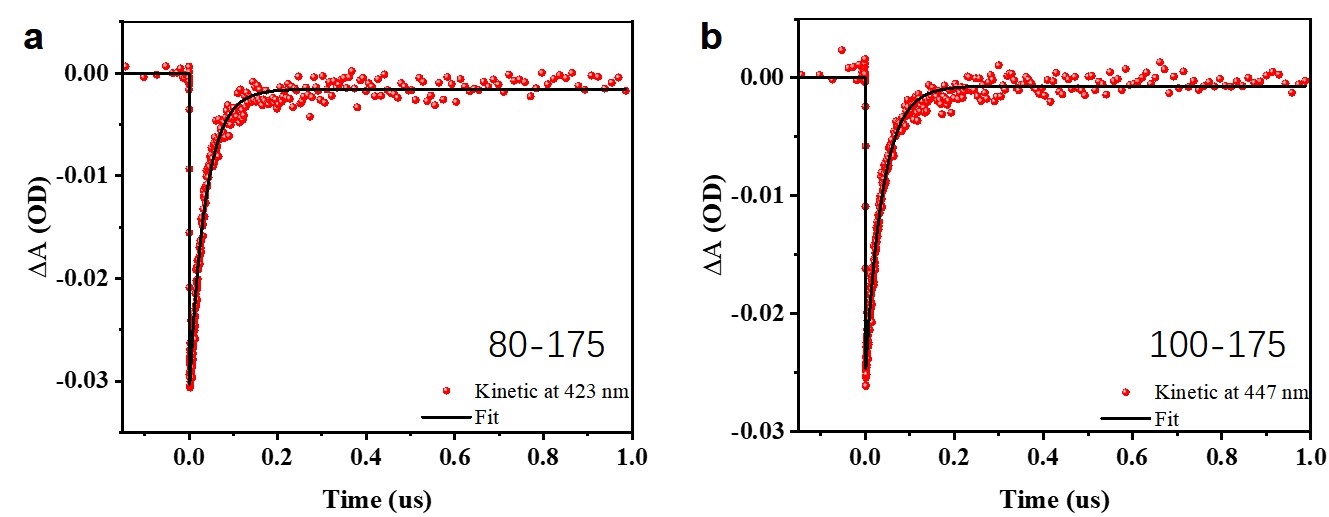


**Fig. S6**. The time-resolved spectra of microseconds TA experiments a, The microseconds TA experiments of Si_3_N_4_-ITO 80 nm-175 nm. b, The microseconds TA experiments of Si_3_N_4_-ITO.

**S7. Relationship between transient reflectance spectra and Si_3_N_4_ thickness**

The biphasic all-optical switching controls the positive and negative aspects of the signal through variations in the thickness of the top Si_3_N_4_ layer. To validate the universality of this approach for the Si_3_N_4_ -ITO system, we prepared a set of samples with ITO thickness of 220 nm and Si_3_N_4_ thickness of 80 nm/100 nm, maintaining identical pumping conditions as described in the main text. The transient reflectance spectra in the vicinity of 324 nm, illustrated in Supplementary Fig. S7a and b exhibit biphasic characteristics for both thicknesses. Specifically, the 80 nm Si_3_N_4_ thickness shows a positive response, while the 100 nm thickness demonstrates a negative response. Their ΔR/R kinetics of Si_3_N_4_, as shown in Supplementary Fig. S7c and d, evidently display consistent switching speeds, modulation depths, and opposite polarities.


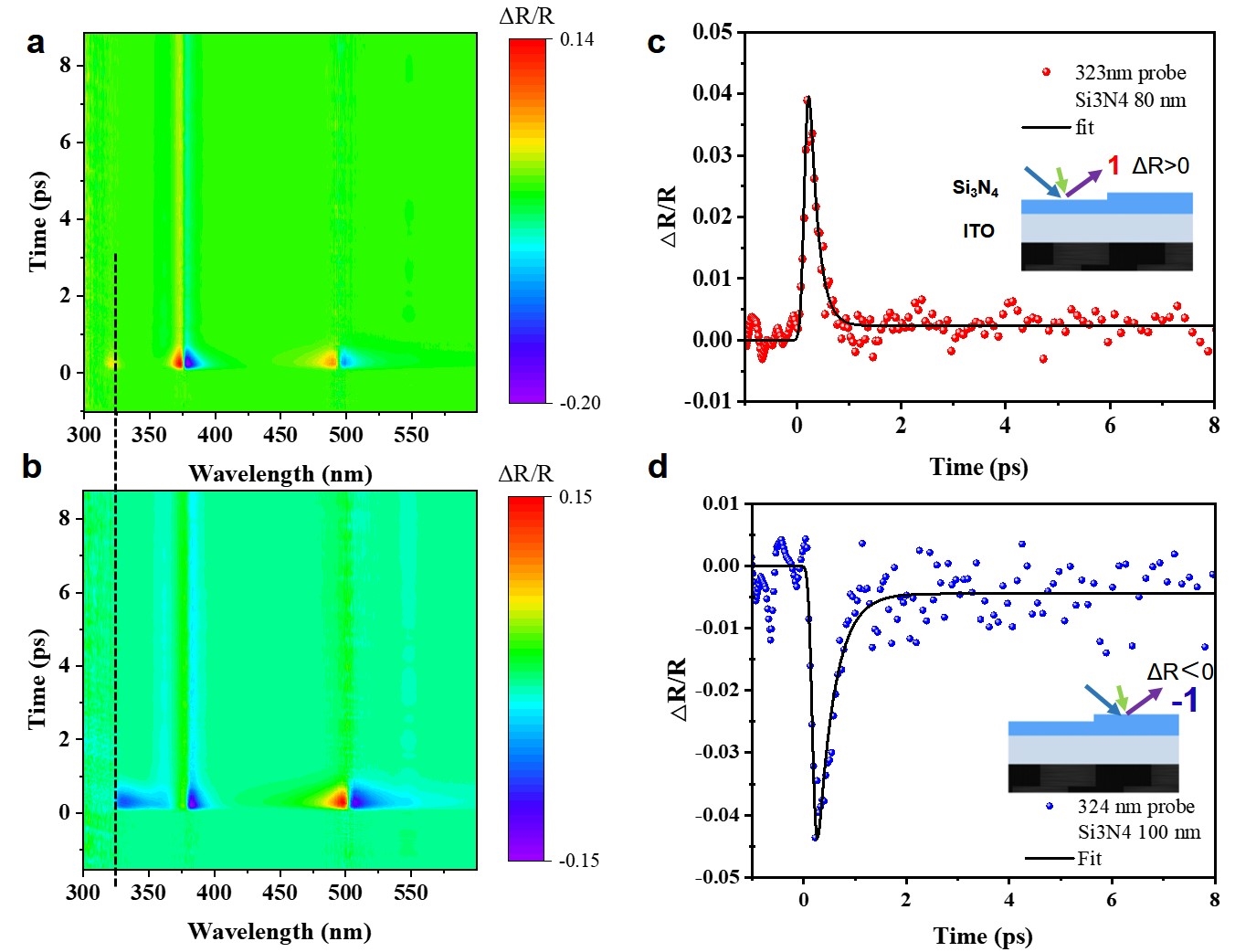


**Fig. S7.** The reproduction of the ultrafast biphasic all-optical switching using a sample with a 220 nm ITO layer. a, The time resolution ΔR/R spectral map of Si_3_N_4_-ITO 80 nm-220 nm. b, The time resolution ΔR/R spectral map of Si_3_N_4_-ITO 80 nm-175 nm. c, The ΔR/R kinetics of Si_3_N_4_-ITO 100 nm-175 nm. d, The ΔR/R kinetics of Si_3_N_4_-ITO 100 nm-220 nm. The pump centre wavelength is kept at 550 nm and pump fluences are 0.43 mJ/cm2, respectively.

We further investigated the impact of Si_3_N_4_ thickness on the transient reflectance spectra. Si_3_N_4_ layers with thicknesses of 60/80/100/120/140 nm were grown on ITO with a thickness of 170 nm. Their transient reflectance spectra are shown in Supplementary Fig. S8a-e. As the Si_3_N_4_ thickness varies from 60 nm to 120 nm, the transient reflectance spectra at 330 nm exhibit a transition from negative to positive and back to negative. When the Si_3_N_4_ thickness reaches 140 nm, the resonance position shifts to 320 nm. The ΔR/R kinetics of Si_3_N_4_ with different thicknesses are presented in Supplementary Fig.S8f-j. Despite the lower signal-to-noise ratio for the 140 nm Si_3_N_4_ thickness, all samples exhibit sub-picosecond switching speeds. These data indicate the versatility of our Si_3_N_4_ -ITO all-optical switching, providing various design options for ultraviolet biphasic all-optical switching.


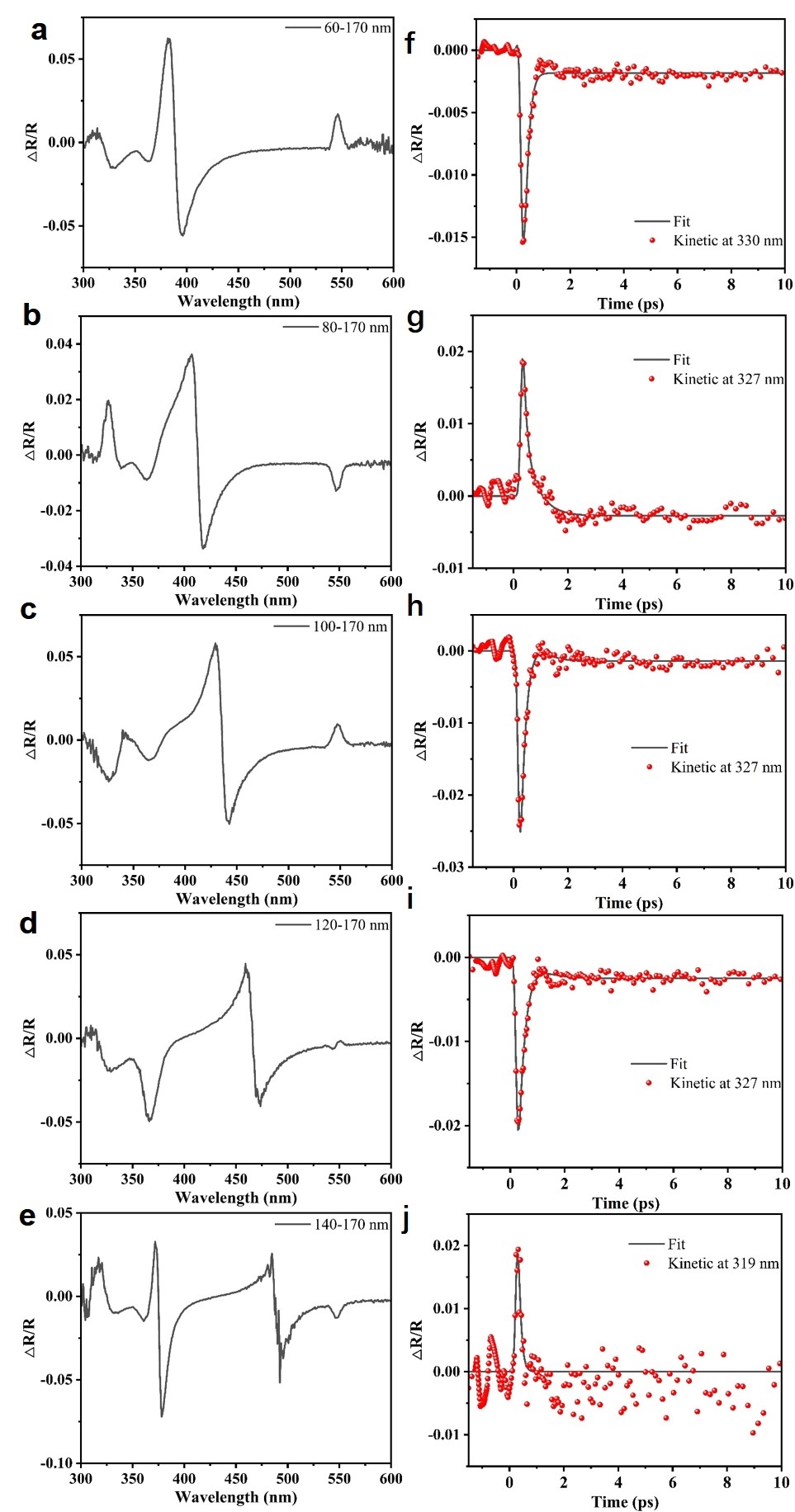


**Fig. S8.** The impact of Si_3_N_4_ layer thickness on transient reflectance spectra. a-e, ΔR/R spectrum of Si_3_N_4_ with a series of different thicknesses. f-j, The ΔR/R kinetics of Si_3_N_4_ with a series of different thicknesses. The pump center wavelength is kept at 550 nm and pump fluences are 0.43 mJ/cm^2^, respectively.

**S8. Power dependence**

In correspondence with the 550 nm pump discussed in the main text, we examined the power dependence under 266 nm wavelength excitation. In the ultraviolet region around 330 nm, the transient reflectance initially increases and then decreases with the increasing pump fluence, exhibiting a saturated trend. In the visible region, the transient reflectance continues to increase with the pump fluence, approaching a nearly linear trend.

**
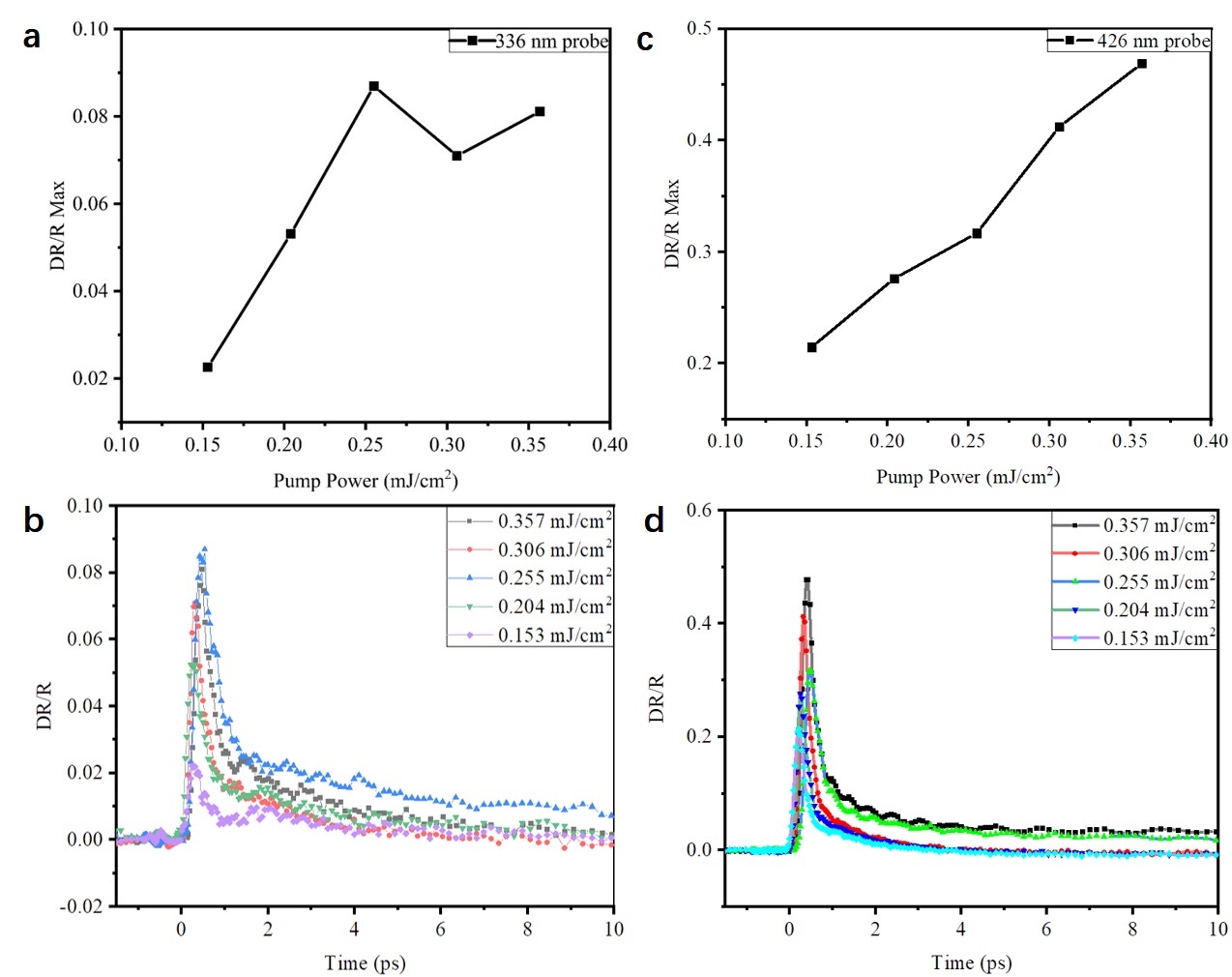
**

**Fig. S9.** The power dependence of the transient reflectance spectra under 266 nm wavelength pump for the device. a, Dependence of modulation depth of ΔR/R(Max) on pump fluence at ultraviolet band. b, Dependence of switching speed on pump fluence at ultraviolet band. c, Dependence of modulation depth of ΔR/R(Max) on pump fluence at visible band. d, Dependence of switching speed on pump fluence at visible band. The pump centre wavelength is kept at 266 nm, respectively.

**S9. Temperature dependence of transient reflectance spectra**

We characterized the temperature dependence of the Si_3_N_4_-ITO all-optical switching, revealing distinct variations in the transient reflectance spectra from the ultraviolet region around 330 nm to the visible region near 425 nm as the temperature varied from 77 K to 310 K. In the ultraviolet region, at low temperatures (77 K and 127 K), the transient reflectance spectra exhibited negative ΔR/R<0, while in the temperature range of 177 K to 310 K, positive ΔR/R values increased, accompanied by a resonance position shift. Conversely, in the visible region around 425 nm, ΔR/R increased positively with temperature, showing no transition from negative to positive values. Consequently, we conclude that our Si_3_N_4_-ITO ultrafast all-optical switching demonstrates temperature dependence, suggesting potential applications in ultrafast temperature sensing. Furthermore, the observed complexity in the resonance modes around 330 nm, influenced by inter-band transitions, reinforces the intricate nature of this anomaly, highlighting significant potential in designing ultraviolet ultrafast all-optical switchings.


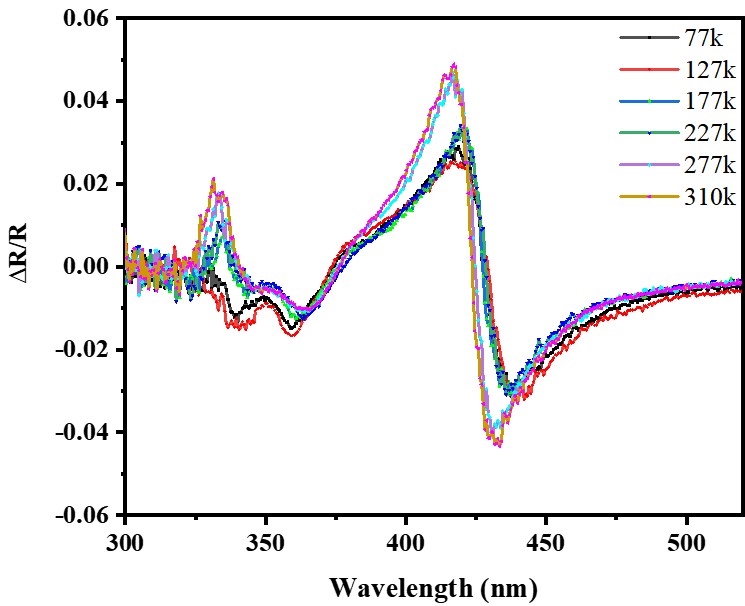


**Fig. S10.** The temperature dependence of the transient reflectance spectra for the device under 550 nm pump

**S10. Schematic diagram of the spectra illustrating the principle of the biphasic all-optical switching**

In Fig. R3(a) and (b), the spectra show the shift of F-P resonance caused by changes in the material's refractive index after pump excitation, without involving interband transitions. This is consistent with the response observed at a wavelength of 425 nm in the manuscript. Fig. R3(c) and (d) depict the effect of F-P resonance superimposed on the interband transition region at 330 nm. Since ITO exhibits strong absorption at wavelengths below 350 nm, the steady-state spectrum displays a broad absorption band with a narrower resonance embedded within it. When the material is pumped and the F-P t resonance shifts to the shorter wavelength region with strong absorption, the transient reflectance spectrum will only exhibit a positive ΔR at 330 nm, while in the shorter wavelength region, ΔR is very small and can be nearly disregarded. Figures R3(e) and (f) show the case of Si3N4 with a different thickness, where the F-P resonance slightly deviates from 330 nm, resulting in a relatively high reflectance at 330 nm. After the pump-induced change in the material's refractive index, a positive ΔR is observed at 330 nm, while ΔR in the shorter wavelength region remains extremely small and can be nearly disregarded. These two different Si_3_N_4_ thicknesses exhibit opposite signs of ΔR in the transient reflectance spectra, thereby forming a biphasic all-optical switching.

**
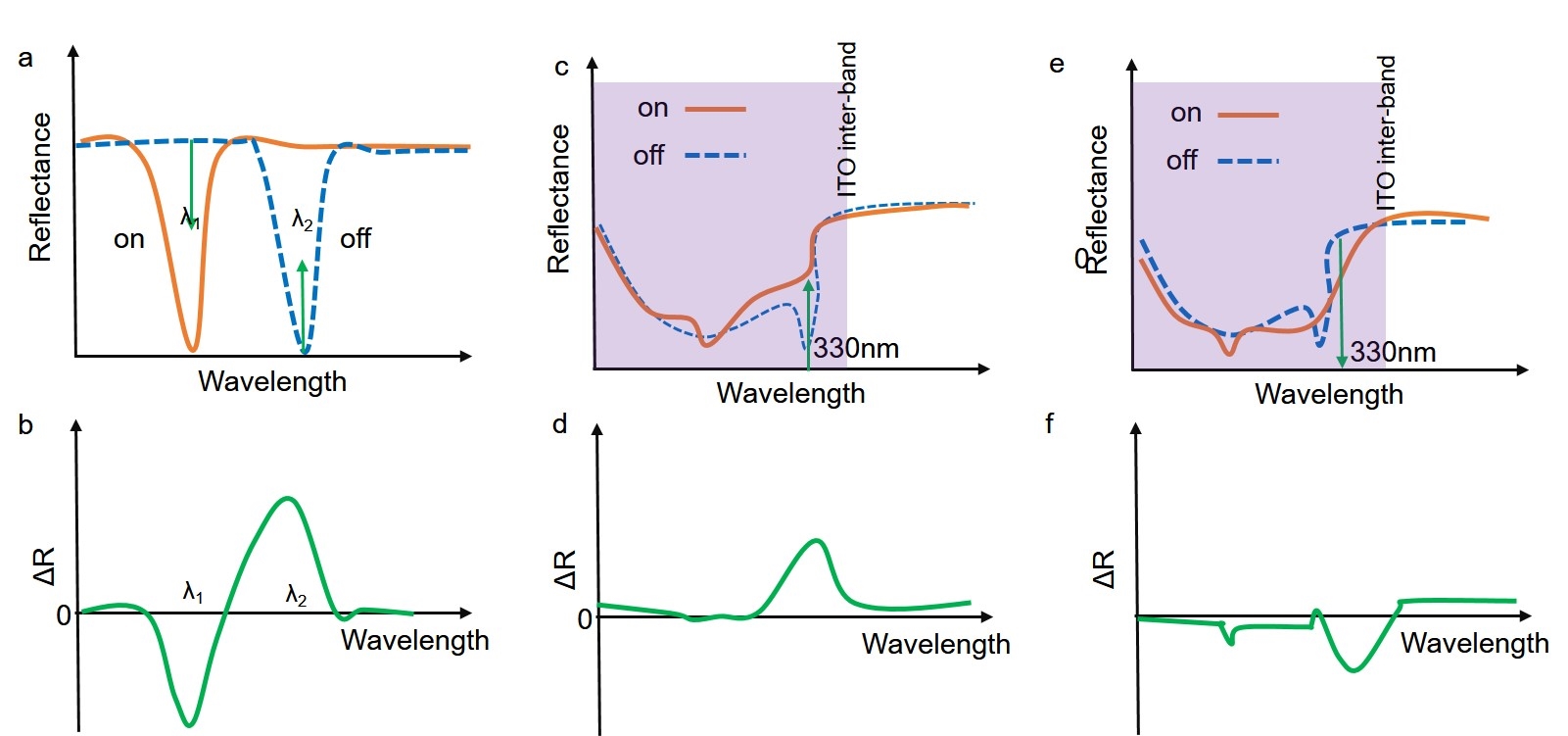
**

Fig.S11. Schematic diagram of the spectra illustrating the principle of the biphasic all-optical switch through the superposition of Fabry-Pérot resonance and interband transitions. a and b show simplified spectra without involving interband transitions. c–f depict the spectral schematic of the biphasic switch for different Si_3_N_4_ layer thicknesses (80/100 nm)

**
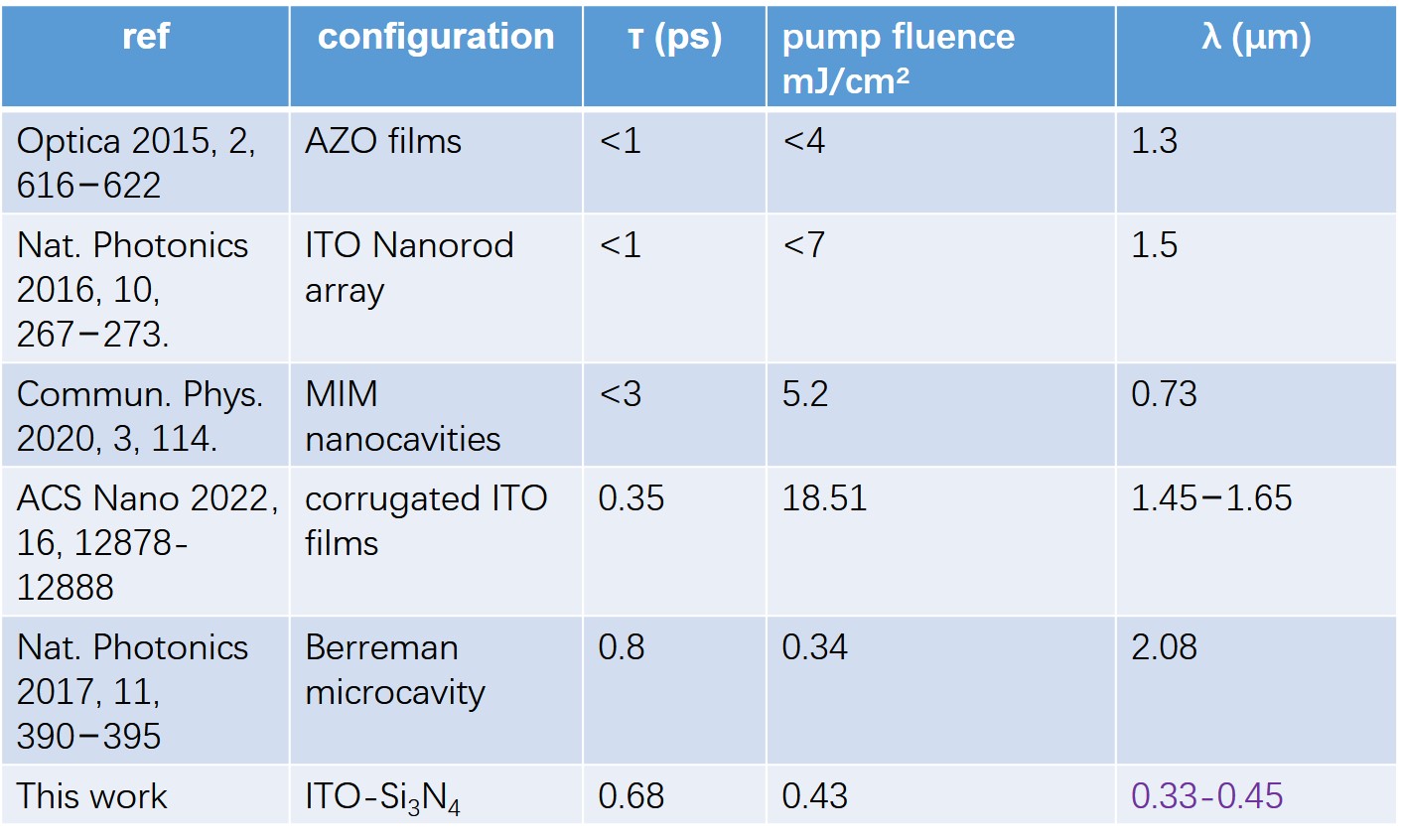
**

**References**

[1] P. Guo, R. D. Schaller, J. B. Ketterson, and R. P. H. Chang, "Ultrafast switching of tunable infrared plasmons in indium tin oxide nanorod arrays with large absolute amplitude," *Nature Photonics,* vol. 10, pp. 267-273, 2016.

[2] Y. Yang, K. P. Kelley, E. Sachet *et al.*, "Femtosecond optical polarization switching using a cadmium oxide-based perfect absorber," *Nature Photonics,* vol. 11, pp. 390-395, 2017.

[3] P. Guo, R. D. Schaller, L. E. Ocola, B. T. Diroll, J. B. Ketterson, and R. P. H. Chang, "Large optical nonlinearity of ITO nanorods for sub-picosecond all-optical modulation of the full-visible spectrum," *Nature Communications,* vol. 7, no. 1, p. 12892, 2016.
